# Faster dense deformable image registration by utilizing both CPU and GPU

**DOI:** 10.1117/1.JMI.8.1.014002

**Published:** 2021-02-01

**Authors:** Simon Ekström, Martino Pilia, Joel Kullberg, Håkan Ahlström, Robin Strand, Filip Malmberg

**Affiliations:** a Uppsala University, Section of Radiology, Department of Surgical Sciences, Uppsala, Sweden; b Antaros Medical, Mölndal, Sweden; c Uppsala University, Centre for Image Analysis, Division of Visual Information and Interaction, Department of Information Technology, Uppsala, Sweden

**Keywords:** Atlas-based segmentation, brain MRI, deformable image registration, graphics processing unit

## Abstract

**Purpose:** Image registration is an important aspect of medical image analysis and a key component in many analysis concepts. Applications include fusion of multimodal images, multi-atlas segmentation, and whole-body analysis. Deformable image registration is often computationally expensive, and the need for efficient registration methods is highlighted by the emergence of large-scale image databases, e.g., the UK Biobank, providing imaging from 100,000 participants.

**Approach:** We present a heterogeneous computing approach, utilizing both the CPU and the graphics processing unit (GPU), to accelerate a previously proposed image registration method. The parallelizable task of computing the matching criterion is offloaded to the GPU, where it can be computed efficiently, while the more complex optimization task is performed on the CPU. To lessen the impact of data synchronization between the CPU and GPU, we propose a pipeline model, effectively overlapping computational tasks with data synchronization. The performance is evaluated on a brain labeling task and compared with a CPU implementation of the same method and the popular advanced normalization tools (ANTs) software.

**Results:** The proposed method presents a speed-up by factors of 4 and 8 against the CPU implementation and the ANTs software, respectively. A significant improvement in labeling quality was also observed, with measured mean Dice overlaps of 0.712 and 0.701 for our method and ANTs, respectively.

**Conclusions:** We showed that the proposed method compares favorably to the ANTs software yielding both a significant speed-up and an improvement in labeling quality. The registration method together with the proposed parallelization strategy is implemented as an open-source software package, deform.

## 1 Introduction

Image registration is an important aspect of medical image analysis and a key component in many analysis concepts. Applications include fusion of multimodal images, multi-atlas segmentation,^1^ and whole-body analysis.^2^ Deformable image registration is generally computationally expensive and implementing methods for registration usually involves a trade-off between the quality of the results and the computation time required to produce them. The need for computationally efficient registration methods is highlighted by the emergence of large-scale image databases, e.g., the UK Biobank,^3^ providing multimodal imaging from 100,000 participants. Extensive reviews on image registration are available.^4^^–^^6^

Dense deformable image registration aims to find the correspondences on a voxel-to-voxel basis. This leads to a resulting displacement field that has the same resolution as the input images, i.e., one displacement vector for each voxel. The popular advanced normalization tools (ANTs) software package with the symmetric image normalization method (SyN)^7^^,^^8^ produces such displacement fields. Other approaches provide very efficient registration but with deformation grids of lower resolution^9^ and even computation times of a few seconds has been achieved.^10^

In our recent work,^11^ we introduced a dense image registration method based on discrete optimization by minimal graph cuts with α-expansion.^12^ The use of minimal graph cuts for image registration has previously been proposed by others^13^^,^^14^ but has seen limited adoption in practice due to the high computational cost of this approach. By dividing the image into subregions and restricting each α-expansion to a single subregion at a time, we were able to drastically reduce the computation time of this registration approach with only a small penalty in terms of quality.

Processing a subregion involves two steps: computing the voxelwise matching criterion (i.e., constructing the graph) and performing discrete optimization by solving a minimal graph cut problem. Early profiling experiments revealed that, for smaller subregions, the majority of the computation time was spent computing the matching criterion, and not in performing the graph cut optimization. This effect was even more pronounced when using more computationally intensive similarity metrics, e.g., cross-correlation (CC), which has been proven valuable in image registration.^15^

The matching criterion can be decomposed as a sum of independent terms over voxels, whose calculation is straightforward to parallelize. We, therefore, propose to further reduce the computational cost of our previously proposed registration method^11^ by moving the computation of the matching criterion to the highly parallel graphics processing unit (GPU), while still performing the graph cut optimization on the central processing unit (CPU). The reasoning is that an optimization task of this nature is generally harder to parallelize and performing all works on the GPU would result in valuable computing resources, i.e., the CPU, not being utilized. A potential bottleneck of this heterogeneous computing approach is the data sharing between processors. To lessen the impact of data synchronization, we propose a pipeline model, effectively overlapping computational tasks with data synchronization. The resulting implementation is then evaluated in a brain labeling task and compared empirically with the ANTs software package. ANTs was chosen due to its proven performance in brain labeling^16^ and the fact that it produces a high-resolution displacement field. Further evaluation of ANTs can be found in the literature.^9^^,^^15^

This work aims to present an efficient parallel computation strategy for GPU accelerated image registration. Both the performance and the quality of the presented implementation are evaluated. The method is implemented as a software package, deform, and made publicly available as an open-source project in an attempt to facilitate further research and development.

## 2 Related Work

The modern GPU and general-purpose computing on graphics processing units (GPGPU) have played a critical role within high-performance computing in the last two decades.^17^ The affordability and high computing power in terms of flop/s are two large contributors to the rapid development of GPGPU. GPU acceleration has been adopted within a large range of fields, including medical image analysis.^18^^,^^19^ They have also been a large contributor to the rapid growth of deep learning.^20^ However, for GPUs, and heterogeneous computing in general, a common challenge is the task of sharing data between the processors. It is not uncommon that the transfer overhead for PCI Express is a large bottleneck for GPU-based applications even though the computing performance is rapidly increasing.^21^ The increased amount of available memory on recent GPUs (e.g., 11 GB on NVIDIA GTX 1080 Ti) makes it easier to avoid the costly transfer for applications that do not need to continuously synchronize data between CPU and GPU. It is also possible to stream data asynchronously over PCI express to minimize the performance loss caused by the transfer. This allows for overlap of computation and transfer operations but at the cost of increased application complexity.

Extensive effort has been put on accelerating the task of image registration using GPUs. Available methods can be grouped into two categories: methods implemented fully on the GPU, and heterogeneous methods, utilizing both CPU and GPU. The reasoning for heterogeneous methods is generally that certain tasks are less trivial to parallelize. Older GPUs also lack efficient double-precision floating-point operations, making them unsuitable for tasks requiring high precision. However, data synchronization quickly becomes an obstacle for methods employing a dense deformation model. The Demons algorithm together with variations has been fully implemented on the GPU, presenting speed-up by factors of 35 to 40 with no loss in accuracy compared with their corresponding CPU implementations.^22^^,^^23^ Mutual information, another computationally expensive metric commonly used for multimodal image registration, has been successfully accelerated by GPUs in several cases^24^^–^^26^ and even real-time performance has been achieved.^27^ A reformulation of the free-form deformation to enable acceleration by GPUs was proposed by Modat et al.^28^ GPU acceleration has also been implemented in the registration package elastix.^29^ Specifically, the Gaussian pyramid computation and the image resampling were accelerated by the GPU. This resulted in acceleration by a factor of 4 to 5 on a machine with eight physical cores. Another efficient heterogeneous approach where the matching criterion was computed on the GPU and the optimization was performed on the CPU was proposed by Ellingwood et al.^30^ This method implemented a composite transformation model to reduce the required CPU-GPU communication and demonstrated a speed-up by a factor of 4. Multiple surveys on the topic of image registration on GPUs have been published.^31^^,^^32^

ANTs do not utilize GPU computing for the image registration but efforts have been put into utilizing the multithreading functionality introduced in Insight Toolkit 4 (ITK).^8^ Implementations of using GPUs to accelerate the similarity metric computation of ANTs have been reported in literature,^33^ but no implementation was publicly available at the time of writing.

## 3 Preliminaries

### 3.1 Efficient Graph-Cut-Based Registration

In this section, we briefly recall our previously proposed efficient graph cut-based registration method.^11^ We define an image I as a pair I=(V,I), where V is the set of voxels on a regular grid and I a mapping I:V→R. We consider registration from a source image, $S=(V_{S},I_{S})$ to a target image, $T=(V_{T},I_{T})$.

Deformations are in this setting represented by a dense grid of displacement vectors, where each point within the image can be displaced arbitrarily. The sought transformation between the two input images can be defined by the mapping $W:R^{3}\to R^{3}$. W maps each voxel in T to a voxel in S. The deformation model is represented by a displacement field, u, where u(x) for $x\in V_{T}$. Thus, W can be defined as W(x)=x+u(x). Throughout, we use trilinear interpolation to define the values of both images and displacement fields at nongrid points.

We phrase registration as an optimization problem and seek a displacement field that minimizes an objective function f in the form

f(u)=D(u)+αR(u), (1)

where D is a data term, measuring the similarity between the target image and the transformed source image, and R is a regularization term that penalizes nonsmooth deformation fields. The user-defined parameter α controls the balance between the two terms. In the experiments presented here, Pearson’s correlation coefficient (PCC) was employed as similarity metric. The PCC is computed independently for each voxel, and the neighborhood is assumed to be rigid with respect to the center voxel. This is due to the registration algorithm considering all voxels and their corresponding displacement vectors individually. PCC can be defined as

$$\mathrm{PCC}(A,B)=\frac{\underset{v\in V}{\sum }(I_{A}(v)-{\overset{¯}{I}}_{A})(I_{B}(v)-{\overset{¯}{I}}_{B})}{\sqrt{\underset{v\in V}{\sum }{\left(I_{A}(v)-{\overset{¯}{I}}_{A}\right)}^{2}\underset{v\in V}{\sum }{\left(I_{B}(v)-{\overset{¯}{I}}_{B}\right)}^{2}}},$$ (2)

where A and B are the two images to match and $\overset{¯}{I}$ denotes the mean value of I over V, V in this case being the shared domain of A and B. The data term can thus be defined as

$$D_{\mathrm{PCC}}(u)=\underset{v\in V_{T}}{\sum }\frac{1}{2}(1-\mathrm{PCC}((\omega (v,r),I_{T}),(\omega (v,r),I_{S}\circ T_{u(v)}))),$$ (3)

where the PCC is computed for patches of T and S defined by small spherical windows, $\omega (v,r)\subseteq V_{T}$, centered in v with radius r. $T_{u(v)}$ is a translation defined by vector u(v). The coefficient ranges from −1 to 1, with values below zero denoting negative correlation, and values above zero denoting positive correlations. In this case we only consider positive correlations but for other purposes, such as multimodal registration, the metric may be reformulated to match negative correlations as well.

For the regularization term, a diffusion regularizer was applied,^34^ defined as

$$R(u)=\underset{(v,w)\in N}{\sum }{\left\|u(v)-u(w)\right\|}^{\gamma }.$$ (4)

Here, N is the set of all pairs of voxels that are adjacent according to the standard 6-neighborhood, and γ is a parameter that affects the strength of the regularization, with higher values of γ implying stronger penalty for high values of the first derivative of the transform, while keeping low penalty for small values of the derivative. The Appendix provides a proof that our binary terms are submodular, a requirement for being able to solve the maximum flow/minimum cut problem in polynomial time.

By constraining the displacement vectors to lie on a regular grid, the problem of finding a u that minimizes the f(u) can be cast as a discrete labeling problem. The grid spacing ϵ is typically selected to be smaller than the voxel spacing in the image, to allow registration with subvoxel precision. An iterative move-making approach is used to find an optimal displacement field u. A move, in this context, consists of changing a given displacement field by moving the displacement vectors at some subset of the voxels by a vector of length ϵ along a specified coordinate axis. This vector can be defined as

$$\delta =\epsilon e_{i},$$ (5)

where $e_{i}$ is a unit vector aligned to one of the coordinate axes. Starting from an arbitrary initial displacement field we determine, at each iteration, if the current displacement field can be improved by performing such a move. The process continues until convergence, e.g., until no move that improves the solution exists. Determining the best possible move along an axis at a given configuration is a binary labeling problem – each voxel either moves ϵ along the given axis or remains unchanged. The space of all binary labelings (i.e., moves along a given axis) is extremely large, and thus an exhaustive search is not feasible. It turns out, however, that an optimal move can be found in low-order polynomial time by solving a minimal graph cut problem.^12^^,^^35^

To find an optimal move we begin by redefining u as a function of the sought binary labeling and δ

$$u^{\prime }(x)=u(x)+L(x)\delta ,$$ (6)

where the binary labeling is defined by the function $L:V_{F}\to \{0,1\}$. Further, we redefine our matching criterion as a function of the same labeling

$$f(u^{\prime })=\underset{v\in V_{F}}{\sum }\phi_{v}(L(v))+\underset{(v,w)\in N}{\sum }\phi_{v,w}(L(v),L(w)).$$ (7)

The unary term, $\phi_{v}:\{0,1\}\to R$, is equal to the data term in Eq. (3),

ϕv(L(v))=∑v∈VT12(1−PCC(T|ω(v,r),S^|ω(v,r))), (8)

where S^ is transformed according to

I^S(v)=IS(v+u(v)+L(v)δ). (9)

Similarly, the binary term, $\phi_{v,w}:\{0,1\}\times \{0,1\}\to R$, is defined according to our regularization term in Eq. (4):

$$\phi_{v,w}(L(v),L(w))={\left\|(u(v)+L(v)\delta )-(u(w)+L(w)\delta )\right\|}^{\gamma }.$$ (10)

In summary, the optimization method starts from an arbitrary initial displacement field (e.g., the identity transform). The displacement field is then iteratively improved by a sequence of moves—each determined by solving a minimal graph cut problem—until no further improvement can be made. To avoid poor local minima, the algorithm is combined with a multiresolution strategy.

Solving a minimal graph cut problem to determine the optimal move across all voxels in the image is computationally expensive, and thus a direct application of the optimization method described above is not practically feasible for registration of large volume images. The main contribution of our previous work^11^ is the observation that considering all voxels at every iteration is not strictly necessary, as interactions between distant voxels are unlikely to significantly affect the result. By dividing the image into smaller subregions and restricting each move to only modify the displacement vectors within one region at a time, the computation time of the method described above can be reduced drastically (from days to minutes) with only a small penalty in terms of quality of the solution.

### 3.2 GPU Programming Model

For the purpose of this paper, only NVIDIA GPU architectures and related nomenclature were considered. At this level of abstraction, other commonly used architectures, e.g., AMD, are not too dissimilar. An extensive overview of modern GPU architectures has been presented by Owens et al.^17^

The modern GPU consists of a number of streaming multiprocessors (SM), each containing a number of streaming processors (SP). Today a typical GPU (NVIDIA GTX 1080 Ti) has 3584 SPs divided among 28 SMs. GPUs are, despite this, very primitive, including only a simple instruction set and very limited control logic. They are thus generally considered a supplement to the more common CPU. The CPU, which together with its memory is referred to as the host, is responsible for scheduling computation resources and transferring data to (upload) and from (download) the GPU and its memory (i.e., the device).

The compute unified device architecture (CUDA) programming model was introduced by NVIDIA to enable general computations on GPU hardware. CUDA employs a single instruction multiple thread model of parallelization. Each thread, mapped to an SP, will execute the same instructions on different data, this can also be referred to as data parallelism. To facilitate concurrency, the concept of streams was introduced, extending the regular data parallelism. A stream is a sequence of operations that are executed in order on the GPU. These operations could be either kernel invocations, i.e., routines executed on the GPU, or transfer operations, e.g., host-device communication. The scheduler will do its best to execute operations in different streams concurrently, i.e., executing kernels on available SM resources or performing asynchronous data transfers. Hence, this stream model promotes task parallelism. An extensive introduction to GPGPU and CUDA has been presented by Sanders and Kandrot.^36^

## 4 Method

This work is an extension to our previous method.^11^ Preliminary evaluations identified the matching criterion computation and the optimization as the two largest time consumers, with a majority of the time being spent on the former. This section presents a strategy where the computation of the matching criterion is performed on the GPU, greatly alleviating the CPU load. Furthermore, a pipeline model is presented to lessen the impact of the required CPU-GPU communication.

**Fig. 1:**
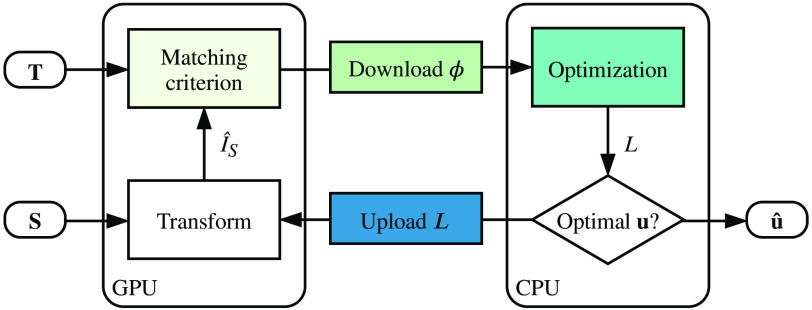
Diagram of the registration loop and the interaction between CPU and GPU. Input volumes, T and S, are directly uploaded to the GPU and u is initialized. S is transformed by u and passed to the matching criterion function. The criterion is computed and the terms, ϕ, are downloaded to the CPU. The CPU performs the optimization and uploads the resulting labeling, L, to the GPU, where it is applied to u. This loop is iterated until u converges.

Figure 1 shows an overview of the proposed optimization loop. The process starts by setting u(x) to an arbitrary initial transform (e.g., identity transform). The input images, T and S, are then permanently uploaded to the device. The process is structured similarly to our previous work, with the exception that the matching criterion and the transformation (i.e., resampling with trilinear interpolation) are computed on the GPU.

Using the subregion heuristic the process produces a set of subregions, $B=\{b_{1},b_{2},\ldots ,b_{n}\}$, for each subiteration. The regions can be processed in arbitrary order, making task parallelism trivial. To accelerate this process, a four-stage process is implemented:

1. Let $V^{\prime }$ be the voxels residing in the current subregion. The unary ($\phi_{v}$) and binary terms ($\phi_{v,w}$) for $v,w\in V^{\prime }$ are computed on the GPU, Eqs. (8) and (10), respectively.
2. Download the terms $\phi_{v}$ and $\phi_{v,w}$ to the host memory.
3. Build the graph representation using $\phi_{v}$ and $\phi_{v,w}$ and perform the max-flow/min-cut optimization. This provides the mapping L(x) for $x\in V^{\prime }$.
4. Upload the label mapping L(x) as produced by Eq. (3) to the GPU.

Two kernels were implemented to compute the energy terms, one for each term. The unary kernel takes the two input images, T and S, as input together with the set $V^{\prime }$ defining which voxels to compute. The kernel computes $\phi_{v}(L(v))$ [Eq. (8)] for every voxel in $V^{\prime }$ in parallel, where L(v)∈0,1. The result is written to a buffer of size $2|V^{\prime }|$. This step also includes a trilinear interpolation of S. The GPU provides built-in support for interpolation but at the cost of loss in precision, with only 8-bit precision for the interpolation coefficients.^37^ For this reason, trilinear interpolation was reimplemented on the GPU to reflect the 32-bit precision of the original CPU implementation.

The binary kernel computes the binary terms, i.e., the edges connecting all voxels in $V^{\prime }$. The kernel takes u, δ, and the set $N^{\prime }$ as input. The binary term, $\phi_{v,w}(L(v),L(w))$ [Eq. (10)], is computed with L(v),L(w)∈{0,1} for all pairs in $N^{\prime \prime }$. The terms for the pairs in $N^{\prime }\setminus N^{\prime \prime }$, $\phi_{v,w}(L(v),0)$, are also computed. The computed terms are written to a buffer of size $4|N^{\prime \prime }|+2|N^{\prime }\setminus N^{\prime \prime }|$.

For the active subregion, $\phi_{v}$ and $\phi_{v,w}$ are transferred to the host. The energy term buffers on the device have corresponding pinned (page-locked) memory buffers on the host. The advantage of using pinned memory is that data transfer between host and devices can be performed asynchronously. The optimization is performed as described in the previous section using the Boykov–Kolmogorov algorithm.^38^ The resulting labeling, L(x) for $x\in V^{\prime }$, is stored to a buffer and asynchronously uploaded to the device.

**Fig. 2:**
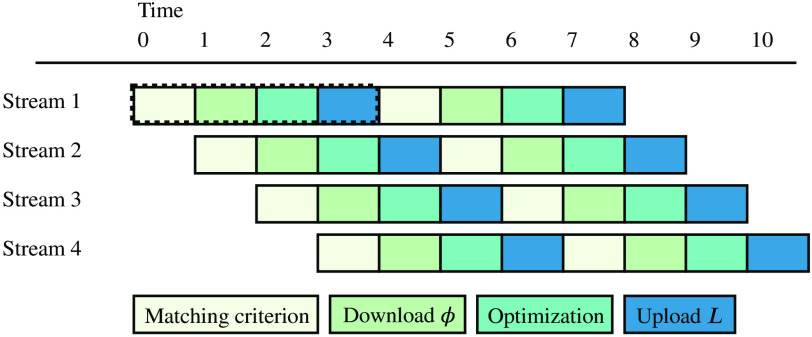
Diagram visualizing the proposed pipeline. Each subregion is processed through four subtasks; computing the matching criterion, downloading ϕ, optimization, and uploading the labeling L. The purpose, as shown, is to overlap the different subtasks in an attempt to increase throughput.

The pipeline is designed to process the subregions in an efficient manner. The key purpose is to allow overlapping data transfers and calculations on both the host and the device. Figure 2 shows the proposed pipeline and how the stages are overlapped between streams. In practice, this was implemented using streams in CUDA. The four processing stages of a subregion are queued to four streams. The optimization stage within the stream pushes the task of optimization to a separate queue and marks the stream as completed. This utilizes the fact that the optimization generally is more time consuming than computing the matching criterion, and the host has more available resources (e.g., 4 streams versus 12 CPU cores). The host processes the queued optimization tasks in parallel, utilizing all available CPU cores. Whenever a stream completes a subregion, a new one is queued until all subregions have been processed.

After all subregions have been processed, the accumulated labels, L(x) for x∈V, are used to update the displacement field u. This is performed by a third kernel, the apply kernel, which takes L(x), δ, and u as input and applies the new displacement, δ, as defined by Eq. (6) for every ∀  x∈V. The results are then stored back into the buffer holding u.

## 5 Experiments

This section describes two experiments performed to evaluate the proposed method.

• First, an empirical comparison between the GPU-accelerated method and its CPU counter-part was performed, testing the assumptions on computation time and resource utilization.
• Second, the quality and computation time of the proposed methods, both CPU-only and the CPU/GPU hybrid, were evaluated in a brain labeling task, using the ANTs software^7^^,^^16^ as a baseline.

Labeling of brain volumes is an important task in neurology and a common approach is to utilize image registration to transfer known labeling from one brain to another. Avants et al.^8^ demonstrated such a task with the purpose of evaluating the ANTs software. This task used a pediatric brain atlas consisting of brain images from 33 two-year-olds with 83 automatically generated regions, as described by Gousias et al.^39^ A similar experiment was performed for this evaluation, using a publicly available atlas of T1-weighted MR volumes of brains and 95 manually segmented regions for 30 adult subjects.^39^^–^^41^

Twenty-five pairs were randomized from a group of 30 subjects, each pair consisting of a target and a source subject. The purpose was to automatically produce the labeling for the target volume from the source labeling by image registration. The source volume was registered to the target volume, generating a displacement field, mapping the source to the target. The produced displacement field was then used to transform the segmented regions from the source volume to the target volume.

Version v0.2 of deform was used for both the CPU and the hybrid CPU-GPU experiments. In addition, both experiments used the same parameter set. Since the deform software does not include any affine registration, ANTs were used to acquire an initial affine transformation. The scripts and parameter files for this experiment were made publicly available. The computation times and resulting displacement fields were collected for each registration.

To further assess the performance of the GPU implementation, the software was profiled using NVIDIA’s nvprof and the NVIDIA Visual Profiler. Only a single randomly selected subject was registered and the mean run times of all four stages were collected for the first iteration. The first iteration of the registration process was measured to capture an approximately equal workload for each subregion. Only the last level of the resolution pyramid was profiled since it was the most time consuming and representative of the total run time.

To evaluate the effect of the available hardware resources on computation time another experiment was performed. In this experiment, the same registration task was performed for 12 different thread configurations on both the CPU and the GPU. All registrations were performed for 1 to 12 number of threads. The number of threads does not directly affect the number of cores that will be utilized, but it limits the number of compute tasks that can be run simultaneously and should thus give an indication of the performance with limited resources.

As a baseline, the same registration task was performed with ANTs version v2.3.1. The ANTs registrations were performed using the script antsRegistrationSyN.sh which is provided together with the ANTs package. This is a three-stage process consisting of rigid, affine, and deformable registration. All stages use a multiresolution strategy with four levels and the shrink factors set to 8×4×2×1, i.e., the resolution is changed by a factor 2 for each level. The rigid and affine stages used mutual information as similarity metric while the deformable stage used SyN together with a CC similarity metric.

ANTs produce a matrix for the affine transform and a displacement field for the deformable transformation, both used to generate the final labeling. For ANTs, the computation time was collected together with the resulting transformation.

The overlap between the ground-truth and the automatically produced segmentations was used to assess the quality of the registrations. The Dice overlap was computed for all 95 regions within all the 25 registered pairs. In addition, the voxelwise Jacobian determinant for u(x), $J_{u}(x)$, was computed. The Jacobian determinant quantifies local volume change, where $J_{u}(x)<1$ implies local contractions and $J_{u}(x)>1$ local expansions. $J_{u}(x)<0$ signals that u inverts, or folds, the space at x, which implies a physically impossible transform. The number of voxels with foldings, $\left|\{x|J_{u}(x)<0\}\right|$, was collected for each resulting displacement field.

All registrations were performed on an Intel Xeon W-2133 with six cores and hyperthreading enabled and an NVIDIA GTX 2080 Ti graphics card.

## 6 Results

The results are summarized in Table 1. The GPU implementation of deform had a mean computation time below 2 min, a speed-up by a factor of 4 when compared with the 7 min of the CPU implementation. The SyN stage of ANTs had a mean computation time of 14 min. The rigid and affine stages, exluded in the table, had a mean computation time of 50.7 (5.85) s. Mean Dice overlap of 0.712 and 0.701 was presented for deform and ANTs respectively with a detected significant difference (p<0.01, two-sided paired t-test). Only a slight difference in the results of the GPU and CPU implementation was observed (0.006% difference in Dice overlap). As for the Jacobian determinant, an average of 36 of voxels with negative Jacobian determinant was observed in each pair for deform and 0 for ANTs.

**Table 1:** Mean (and standard deviation) of computation time, Dice overlap, and number of voxels with negative Jacobian determinant.

Method	Time (s)	Dice	$\left|\{x|J_{u}(x)<0\}\right|$
Deform	419 (34)	0.712 (0.1)	34.6
Deform (GPU)	110 (11)	0.712 (0.1)	36.2
ANTs (SyN)	826 (48)	0.701 (0.1)	0

Table 2 further summarizes the GPU implementation and the time spent per subregion on each stage. It was noted when inspecting the implementation, using the profiler, that the GPU did indeed overlap data synchronization and computational tasks. The GPU also managed to execute multiple kernels for the matching criterion in parallel.

**Table 2:** Average time spent in each iteration and average time spent per subregion for the matching criterion computation, the optimization, and the data synchronization.

Task	CPU	CPU-GPU
Iteration (s)	18.54	3.95
Matching criterion (ms)	8.44	0.13
Optimization (ms)	1.03	1.41
Download ϕ (ms)	N/A	0.025
Upload L (ms)	N/A	0.004

A total of 23,400 subregions were processed in each iteration.

**Fig. 3:**
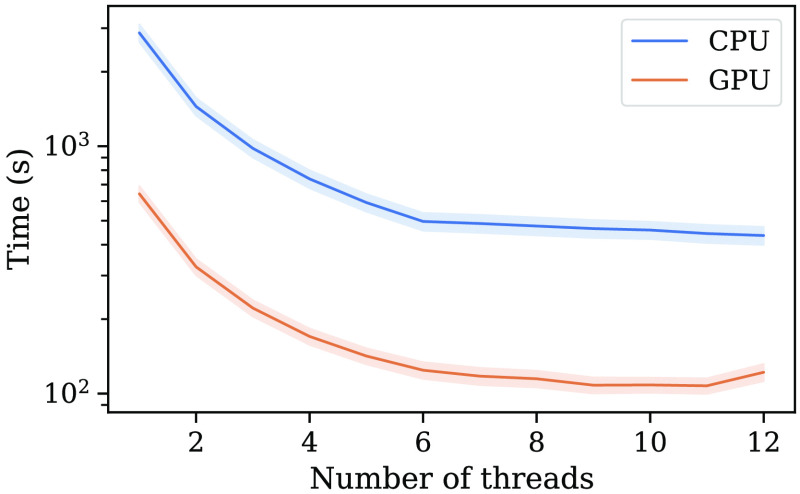
Line plot of the mean runtime for the different thread configurations on the CPU and the GPU implementation of the method. The inner line displays mean runtime and the outer displays standard deviation.

Figure 3 shows the results of the performance analysis when running the registration on different number of CPU cores. Displayed is the mean runtime of the 25 registrations for the different thread configurations.

**Fig. 4:**
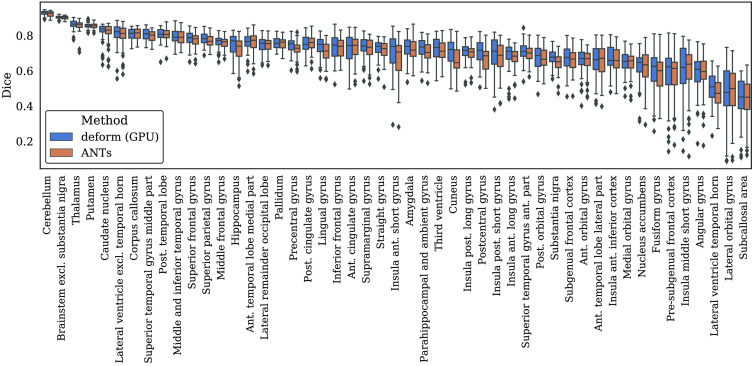
Boxplots of measured Dice overlaps in all regions for the 25 registered pairs. The scores are grouped by method and region, with left and right hemispheres merged to improve visualization. All regions are sorted by median Dice overlap for deform.

Figure 4 shows the Dice overlap for all regions measured in the 25 registered pairs. Regions located both in left and right hemispheres were merged to a single region for visualization. As seen in Table 1, both the CPU and the CPU-GPU version of deform were faster than ANTs.

**Fig. 5:**
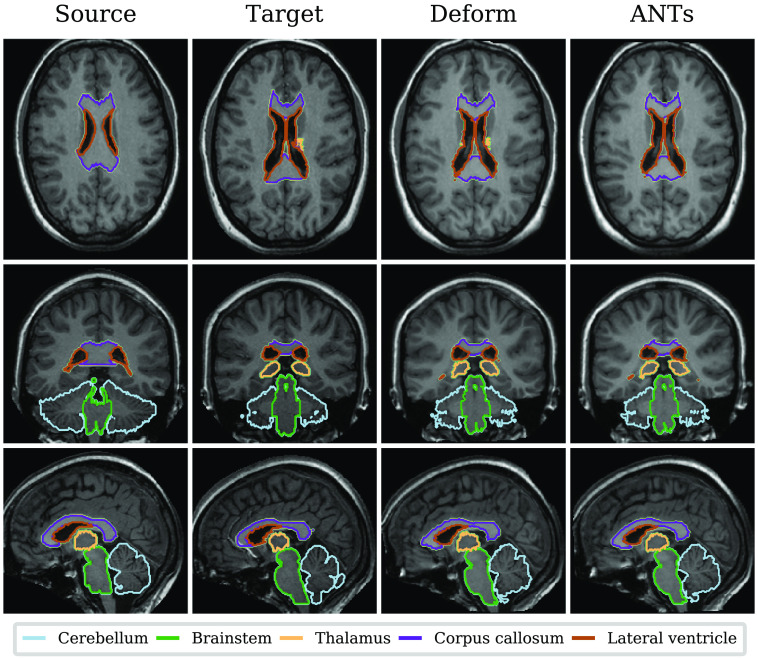
Illustration of a single registered brain pair. Slices from the T1-weighted image and contours of five regions (cerebellum, brainstem, thalamus, corpus callosum, and lateral ventricles excluding temporal horns) are displayed for the input (source and target) and the results of both methods.

Figure 5 shows one randomly selected pair out of the 25 registered brain pairs, showing slices from the T1-weighted images and contours of five common regions selected from the full set of 95. Presented from left to right are the source image, target image, and the resulting images produced by deform and ANTs.

## 7 Discussion and Conclusion

This paper presented an efficient GPU-accelerated implementation of a dense image registration method. The task of computing the matching criterion was offloaded to the GPU, where it could be computed efficiently, while the less trivial optimization task was performed on the CPU. A pipeline was proposed to allow parallelization of all the subtasks, effectively overlapping matching criterion computation, data synchronization, and optimization.

The implementation was first compared with its CPU counterpart. A speed-up by a factor of 4 was observed, which is in line with the results achieved by other heterogeneous methods in the relevant literature. The small differences observed in the resulting displacement fields were negligible and likely caused by floating-point errors when computing the matching criterion. It was observed that around 0.7 s were spent on data synchronization in each iteration (around 18%), but this had a negligible impact on the performance since the pipeline overlapped data transfer and other tasks efficiently. Importantly, it was observed that the matching criterion computation was no longer the bottleneck, as compared with the CPU implementation. Data not reported in this paper showed that changing the regularization parameters within a range were the registration still produced a reasonable result did not affect the presented performance benefit. The benefits diminished when applying less intricate similarity metrics however, such as the sums of squares metric. There was a noticeable time increase in the optimization task (37%), explained by the need for building the graph representation. The CPU implementation had no need for intermediate storage of the graph weights and would build the graph directly in connection to computing the matching criterion.

In the experiments, subregions of size $16^{3}$ were used. Ekström et al.^11^ discussed the effect of the subregion size on the graph cut minimization. Similarly, the subregion size is an important aspect to consider when discussing performance on the GPU. The CUDA block size is an important parameter that may determine the ability to saturate the available SMs. There is a clear connection between task granularity and the possibility to run a task in parallel. However, kernel invocations and data transfers on GPU are not free of overhead and this is an aspect that requires further exploration.

The effect of the number of threads was also investigated to give an indication of the performance when having limited resources. As expected, the computation time decreased as the number of threads were increased. However, this benefit was diminished after around six threads. This could have multiple reasons including the coarse granularity of the tasks or the efficiency of hyperthreading. The benefits of threading are highly dependent upon the ability to divide a task into smaller subtasks. The subtasks are, in this case, the subregions and if the subregions are large enough with respect to the image data we would have more compute threads than blocks resulting in idling threads. Another important aspect is also the fact that the hardware used for these experiments in reality only has six CPU cores with hyperthreading. Hyperthreading simulates additional cores by allowing existing cores to work on two tasks simultaneously. For this reason, the performance benefit cannot be expected to be on the same level as additional hardware cores. Knowing from the previous experiment that the method is bound by the optimization tasks, a change of the GPU hardware would most likely have less, or no, impact on the computation time.

In the second part of the experiment, the proposed method was compared with the popular ANTs software package in the task of labeling brain images. A significant difference in Dice overlap was detected with mean Dice overlap of 0.712 and 0.701 for deform and ANTs, respectively. Figure 4 shows a correlation in the regionwise Dice overlap between the two methods. Although both methods produced results of similar quality, a large difference in computation time was measured for ANTs when compared with both versions of deform. One downside of deform is the lack of guarantees on producing diffeomorphic deformations, but only an average of 38 voxels with a negative Jacobian determinant (0.0006%) was detected in the resulting deformations. This indicates that the regularization term used should be sufficient for reliable results. Increasing the spatial regularization would reduce the number of foldings but also the Dice overlap.

For the future, the task of improving the registration quality mostly involves selecting the best matching criterion and accompanying parameters. This would also be sensitive to the data to register. There are interesting directions for improving performance, however, the most evident direction would be to investigate moving the optimization task to the GPU. There have been efforts to perform graph-cut optimization on GPU, the most prominent attempt being JF-Cut.^42^ However, the practical benefit of such an approach is unclear without further investigation. There would be no data synchronization needed but the GPU would effectively be saturated by both the matching criterion computation and the optimization while the available CPU resources would be unused.

In conclusion, we have presented a parallel computing strategy to efficiently accelerate the task of image registration using GPUs. The method demonstrated a speed-up by a factor of 4 when compared with its CPU-only counterpart. We evaluated the method on a brain labeling task where we compared it with the popular software package ANTs. Our method outperformed the baseline both in terms of quality and computation time. A significant improvement in Dice overlap and a speed-up by a factor of 1.8 and 7.5 were observed for CPU and CPU-GPU versions, respectively. The method presented in this paper is also available in its full as an open-source project.

## 8 Appendix: Proof of Submodularity

A globally optimal solution to the binary labeling problem given in Eq. (7) can be found by solving a maximum flow/minimum cut problem, provided that all binary terms are submodular.^35^ In this appendix, we prove that the binary terms in Eq. (7), given by Eq. (10), are submodular for any u and δ, and for any γ≥2.

A binary term $\phi_{v,w}(L(v),L(w))$ is submodular if it satisfies the inequality

$$\phi_{v,w}(0,0)+\phi_{v,w}(1,1)\leq \phi_{v,w}(0,1)+\phi_{v,w}(1,0).$$ (11)

Here, we have

$$\phi_{v,w}(0,0)={\left\|u(v)-u(w)\right\|}^{\gamma },$$ (12)

$$\phi_{v,w}(1,1)={\left\|(u(v)+\delta )-(u(w)+\delta )\right\|}^{\gamma },$$ (13)

$$\phi_{v,w}(1,0)={\left\|(u(v)+\delta )-u(w)\right\|}^{\gamma },$$ (14)

$$\phi_{v,w}(0,1)={\left\|u(v)-(u(w)+\delta )\right\|}^{\gamma }.$$ (15)

Let γ=2p. We then obtain:

$$\phi_{v,w}(0,0)={\left({\left\|u(v)-u(w)\right\|}^{2}\right)}^{p},$$ (16)

$$\phi_{v,w}(1,1)={\left(\|(u(v)+\delta )-(u(w)+\delta )\|\right)}^{p},$$ (17)

$$\phi_{v,w}(1,0)={\left({\left\|(u(v)+\delta )-u(w)\right\|}^{2}\right)}^{p},$$ (18)

$$\phi_{v,w}(0,1)={\left({\left\|u(v)-(u(w)+\delta )\right\|}^{2}\right)}^{p}.$$ (19)

As presented by Malmberg and Strand,^43^ $\phi_{v,w}$ is submodular for any p≥1 if the following conditions hold:

1. $\phi_{v,w}$ is submodular for p=1.
2. The following inequality holds for p=1:
  $$\max\{\phi_{v,w}(0,0),\phi_{v,w}(1,1)\}\leq \max\{\phi_{v,w}(1,0),\phi_{v,w}(0,1)\}.$$ (20)

A proof that condition 1 holds was given by Ekström et al.^11^ To complete the proof, we thus only need to show that condition 2 holds as well.

Let p=1. Noting that $\phi_{v,w}(0,0)=\phi_{v,w}(1,1)$, the left-hand side of Eq. (20) can be rewritten as

$$\max\{\phi_{v,w}(0,0),\phi_{v,w}(1,1)\}={\left\|u(v)-u(w)\right\|}^{2}.$$ (21)

For the right-hand side of Eq. (20), we may rewrite $\phi_{v,w}(1,0)$ as

$$\phi_{v,w}(1,0)={\left\|u(v)-u(w)\right\|}^{2}+{\left\|\delta \right\|}^{2}+2(u(v)-u(w))\cdot \delta .$$ (22)

Similarly, we may rewrite $\phi_{v,w}(0,1)$ as

$$\phi_{v,w}(0,1)={\left\|u(v)-u(w)\right\|}^{2}+{\left\|\delta \right\|}^{2}-2(u(v)-u(w))\cdot \delta .$$ (23)

We observe that ${\left\|\delta \right\|}^{2}$ is non-negative, and that the dot product 2(u(v)−(u(w)))·δ may be either negative or non-negative. Thus, at least one of the real numbers ${\left\|\delta \right\|}^{2}+2(u(v)-(u(w)))\cdot \delta$ and ${\left\|\delta \right\|}^{2}-2(u(v)-u(w)\cdot \delta )$ are non-negative. Thus, $\max\{\phi_{v,w}(0,0),\phi_{v,w}(1,1)\}\leq \max\{\phi_{v,w}(1,0),\phi_{v,w}(0,1)\}$. This completes the proof.

## Data, Materials, and Code Availability

Soure code for the presented method and the experimental setup is publicly available at https://github.com/simeks/deform and https://github.com/simeks/deform-eval respectively. The imaging data used in the evaluation is publicly available at https://brain-development.org/ (©2007 Imperial College of Science, Technology and Medicine; all rights reserved).
